# Cas9 cleavage assay for pre-screening of sgRNAs using nicking triggered isothermal amplification†
†Electronic supplementary information (ESI) available: Sequence information, reaction conditions optimization and Cas9 cleavage analysis in a time dependent manner. See DOI: 10.1039/c6sc01355d


**DOI:** 10.1039/c6sc01355d

**Published:** 2016-04-29

**Authors:** Kaixiang Zhang, Ruijie Deng, Yue Li, Ling Zhang, Jinghong Li

**Affiliations:** a Department of Chemistry , Analysis Center , Tsinghua University , Beijing 100084 , China . Email: jhli@mail.tsinghua.edu.cn

## Abstract

CRISPR/Cas9 is a highly efficient genome engineering tool, yet its off-target effects and sequence-dependent cleavage activity across different sgRNAs remain major concerns for its application. Here, we propose a nicking triggered exponential amplification reaction (NTEXPAR), a fast and sensitive *in vitro* method, to detect the double strand DNA cleaved by down to 10 pM Cas9 with a linear range of 100 pM to 20 nM. With this newly developed amplification method, Cas9 cleavage activity can be quantified in 40 min and the optimal sgRNA design for specific target sequence can be successfully determined. Using the pre-screened sgRNA, we are able to distinguish single nucleotide mismatch in a gene silencing experiment. This fluorescence based isothermal assay provides a versatile tool for the pre-screening of sgRNAs to achieve highly specific and highly efficient CRISPR/Cas9 genome editing.

## Abstract 2

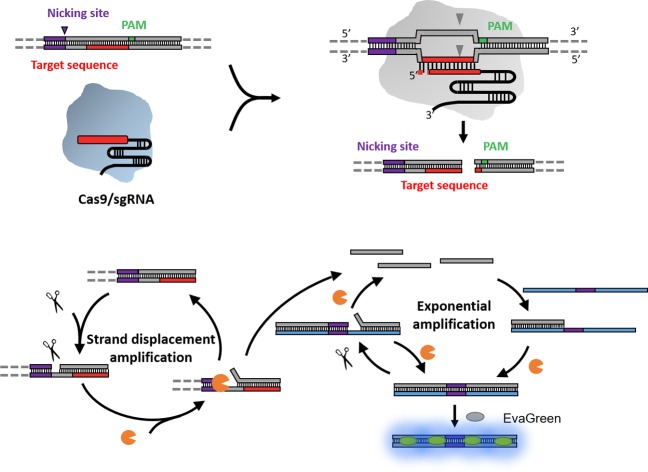
A novel Cas9 cleavage assay was developed for quantitative evaluation of Cas9 cleavage efficiency and pre-screening of sgRNA to achieve highly specific and highly efficient CRISPR/Cas9 genome editing.

## Introduction

Clustered regularly interspaced short palindromic repeat (CRISPR) is an RNA-mediated adaptive immune system which can be programmed with a single guide RNA (sgRNA) to generate site-specific double strand DNA (dsDNA) breaks.^1^,^2^ The CRISPR/Cas9 system has been introduced into mammalian cells as a powerful genome engineering tool for various purposes, including genome editing,^3^ genetic screening,^4^ RNA display^5^ and gene therapy.^6^,^7^ Notably, undesired alterations can be induced at off-target sites by CRISPR which hinder the use of Cas9-based reagents.^8^ To circumvent this problem, Fu *et al.* demonstrated that off-target effects can be dramatically decreased by using truncated sgRNA.^9^ However, the optimal sgRNA design for different targets is variable^10^ and designing sgRNA with a computational model may not be completely reliable because the design criteria are based on limited data.^11^ Therefore, a fast and sensitive *in vitro* Cas9 cleavage assay is in high demand, both for experimental validation of sgRNAs before practical application and minimizing wasted animals on sgRNAs with poor activity.

Gel electrophoresis has served as a standard *in vitro* method for Cas9 cleavage analysis.^12^ Although it is a robust and powerful technology,^13^ it has shortcomings such as being time-consuming, relatively low-throughput (comparing with solution based assays) and the need for radioisotopic labelling to get high sensitivity.^14^,^15^ For example, agarose gel is semi-quantitative at best and quantification relies on band density which leads to a limit of detection in the nanogram range. Although radioactive labelling can increase sensitivity, the inconvenience of labelling makes the whole process take more than 4 h. Polymerase chain reaction (PCR) based methods have also been applied for Cas9 cleavage analysis.^16^ But amplification is always followed by gel electrophoresis or sequencing for readout and usually applied for somatic activity analysis.^17^

Fluorescence-based isothermal amplification has been widely used as a fast and sensitive *in vitro* method for enzyme activity analysis.^18^–^25^ For instance, the exponential isothermal amplification of telomere repeat (EXPIATR) assay has been demonstrated for telomerase activity analysis and achieved single cell level sensitivity.^20^,^26^–^28^ Most conventional isothermal amplifications, such as exponential amplification reaction (EXPAR),^22^ loop-mediated isothermal amplification (LAMP),^29^ strand displacement amplification (SDA)^30^ and rolling circle amplification (RCA),^31^ require a single strand DNA (ssDNA) to trigger a downstream reaction. However, Cas9 cleaved dsDNA only provides a newly formed blunt end at the cleavage site which could not be directly used for isothermal amplification. To solve this issue, we have designed a dsDNA substrate with a nicking site to achieve ssDNA trigger generation for Cas9 cleaved dsDNA detection. Using this design, we first introduce isothermal amplification to Cas9 cleavage efficiency analysis.

Herein, an easy, quick and cost effective fluorescence-based isothermal assay is developed for the analysis of Cas9 cleavage efficiency and pre-screening of sgRNAs. Nicking triggered exponential amplification reaction (NTEXPAR) is demonstrated to be an eligible method for detection of Cas9 cleaved dsDNA. Moreover, it shares the advantages of EXPAR such as being fast, highly sensitive, label free, and abandoning complicated thermal cycling protocols. The dsDNA substrate cleaved by down to 10 pM Cas9 can be successfully detected and the Cas9 concentration can be quantified in a linear range from 100 pM to 20 nM in 40 min. Furthermore, the optimal sgRNA design for specific targets can be successfully determined with this pre-screening process. We have applied the pre-screened sgRNA in a Cas9 mediated gene silencing experiment and we find it can significantly improve the specificity of Cas9 based genome editing.

**Scheme 1:**
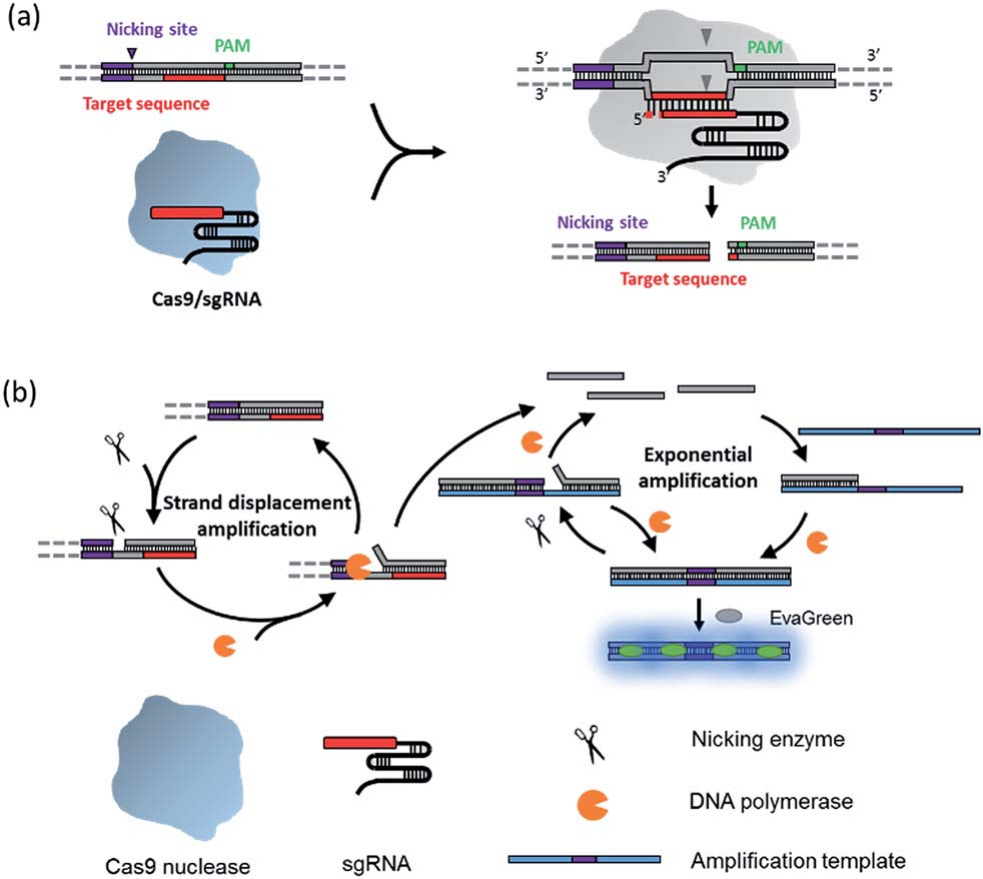
Schematic diagram of the nicking triggered exponential amplification reaction (NTEXPAR) for the detection of Cas9 cleaved dsDNA. (a) The NTEXPAR probe designed for Cas9 cleavage assay contains a nicking site, target sequence and protospacer adjacent motif (PAM) which can be recognized and cleaved by a Cas9–sgRNA complex. (b) After Cas9 cleavage, the exposed blunt-ended cleavage site can be recognized by a nicking enzyme and DNA polymerase to generate a short single-stranded DNA trigger by a nicking and strand displacement mechanism. The generated ssDNA trigger could be further exponentially amplified with the same enzymes in the presence of a designed amplification template. The amount of cleaved dsDNA could be quantified by measuring the product related fluorescence signal.

The principle of the NTEXPAR based Cas9 cleavage assay for pre-screening of sgRNA is illustrated in Scheme 1. The dsDNA substrate is designed with a nicking site, target sequence and protospacer adjacent motif (PAM) which can be recognized by a Cas9–sgRNA complex. The nicking site is designed for ssDNA trigger generation and the PAM is essential for Cas9 recognition.^13^ After Cas9 cleavage, the cleaved dsDNA could be recognized by Nt.BstNBI nicking enzyme and Vent (exo-) DNA polymerase to continuously generate a short ssDNA trigger, while the uncleaved dsDNA substrate could only produce long ssDNA and cannot trigger downstream exponential amplification. The amplification template is designed with two repeated sequences (complementary to the trigger DNA) separated by a nicking site. The generated short ssDNA trigger is able to hybridize with the amplification template and initiates polymerization along the template in the presence of Vent (exo-) DNA polymerase. The newly formed dsDNA is recognized by the nicking enzyme and initiates another nicking and strand extension/displacement cycle. Since the displaced sequence is the same as the trigger ssDNA, it can serve as a free primer to start a new amplification reaction with other amplification templates, eventually leading to exponential amplification and production of a large amount of dsDNA. A dsDNA-binding dye (EvaGreen®) binds to the amplified sequences to generate a fluorescence signal which can be monitored in real time.

## Experimental section

### Materials and apparatus

All oligonucleotides were purchased from Shanghai Sangon Biological Engineering Technology & Services Co., Ltd (Shanghai, China). The dNTPs mix, Vent (exo-) DNA polymerase (2000 U mL^–1^), Nt.BstNBI nicking endonuclease (10 000 U mL^–1^), Cas9 nuclease *S. pyogenes* (1000 nM), HiScribe™ T7 Quick High Yield RNA Synthesis Kit, 10× NEBuffer 3.1 and 10× ThermoPol buffer were purchased from NEB (New England Biolabs, Ipswich, MA, USA). RiboLock RNase inhibitor (40 U μL^–1^) and 50× ROX reference dye were purchased from Life Technologies (Carlsbad, CA, USA). EvaGreen® DNA staining dye and GelRed Nucleic Acid Gel Stain dye were purchased from Biotium (Hayward, CA, USA). CFX 96 real-time PCR detection system (Bio-Rad) was used for controlling reaction temperature and measuring real-time fluorescence signal from exponential amplification reaction (EXPAR) for target DNA detection. A NanoDrop 2000 UV-vis spectrophotometer was used for measuring nucleic acid concentration. A Leica TCS SP5 laser scanning confocal microscope was used for fluorescence imaging.

### *In vitro* transcription of single guide RNA (sgRNA)

Single guide RNA was transcribed *in vitro* with HiScribe™ T7 Quick High Yield RNA Synthesis Kit (purchased from NEB) according to the manufacturer's protocol. Briefly, the transcription templates encoding a T7 promoter and sgRNA were prepared by PCR amplification using a pSpCas9 (BB)-2A-GFP (PX458) plasmid as template. All the primers used in this study are listed in Tables S1 and S4.† The PCR product was purified using an EZ spin column DNA gel extraction kit, followed by resuspension in DEPC H_2_O. The *in vitro* transcription reaction was performed at 37 °C for 6 h with 2 μg of template DNA, 10 μL of NTP buffer mix (5 mM each NTP), 2 μL of T7 RNA polymerase mix and DEPC H_2_O to make the total reaction volume 20 μL. The transcribed sgRNA was purified by Trizol and redissolved in DEPC H_2_O. The concentration of the purified sgRNA was measured using a NanoDrop 2000. After purification, the sgRNA was diluted to a concentration of 1 μM and stored at –80 °C.

### *In vitro* Cas9 cleavage

Cas9–sgRNA complexes were constituted before cleavage by incubating Cas9 nuclease, *S. pyogenes* (purchased from NEB) and the *in vitro* transcribed sgRNA for 10 min at 37 °C in reaction buffer. Cleavage assays were conducted in a reaction volume of 20 μL with 20 nM Cas9 nuclease, 40 nM sgRNA, 1 nM dsDNA substrate and 1 U μL^–1^ Ribolock RNase inhibitor in 1× Cas9 nuclease reaction buffer at 37 °C for 10 min. The cleaved dsDNA was analyzed using NTEXPAR and gel electrophoresis.

### NTEXPAR for Cas9 cleaved dsDNA detection

The NTEXPAR reaction mixture was prepared separately on ice in two parts, part A and part B. Part A consisted of Cas9 cleaved dsDNA, 1× NEBuffer 3.1, 0.2 μM amplification template and 500 μM dNTPs. Part B consisted of 2× ThermoPol buffer, 0.75 U μL^–1^ Nt.BstNBI nicking endonuclease, 0.1 U μL^–1^ Vent (exo-) DNA polymerase, 2× EvaGreen® and 2× ROX reference dye. Part A and part B were pre-heated at 55 °C and mixed immediately before being loaded into a real-time PCR instrument. NTEXPAR was performed at 55 °C and real-time fluorescence intensity was monitored at intervals of 30 s for 40 min using a CFX 96 real-time PCR detection system. A blank control was performed with all the reaction components except the dsDNA substrate. The uncleaved sample was performed without adding Cas9 nuclease. All experiments were performed in triplicate.

### Synthesis of the 3.8 kb DNA long substrate (LS)

To make a head to head comparison between gel electrophoresis and NTEXPAR, a 3.8 kb dsDNA substrate (LS) was synthesized and used for Cas9 cleavage. The 3.8 kb dsDNA substrate (LS) was prepared by PCR amplification using a plasmid containing a Nt.BstNBI nicking site, target sequence and PAM (5′-GAGTC GAAG TAGTGATT T GAG GTA GTA GGT TGT ATA GTT CGC TGG-3′) as a template. The amplified products were purified using an EZ spin column DNA gel extraction kit.

### Gene silencing experiment

The pDsRed2-N1 plasmid and pSpCas9 (BB)-2A-GFP (PX458) plasmid (purchased from Addgene) were used for gene silencing experiments. The procedure of cloning sgRNA into the pSpCas9 (BB) vector was based on a reported protocol.^29^ Briefly, the sgRNA oligo inserts was first phosphorylated by PNK and diluted to a final concentration of 50 nM with DEPC H_2_O. The ligation reaction was performed with 100 ng of pSpCas9, 5 nM phosphorylated oligo inserts, 0.5× T4 ligase buffer, 0.5× Fast digest buffer, 0.1 U μL^–1^ BbsI and 0.05 U μL^–1^ T4 ligase with a total reaction volume of 20 μL. The synthesized plasmid was transformed into an *E. coli* strain and validated by sequencing.

Hela cells were seeded at ∼10 000 cells per well into 24-well plates (BD Falcon) and incubated overnight in DMEM growth media supplemented with 10% FBS and 2% penicillin/streptomycin at 37 °C in 5% CO_2_. Transfections were performed with 300 ng of each plasmid using 1.5 μL Lipofectamine 2000 transfection reagent (Invitrogen) in 600 μL Opti-Mem media (Invitrogen) at 37 °C for 5 h. After 5 h, the Opti-Mem transfection mixtures were removed from the cells and replaced with DMEM growth media for 48 h incubation. Then, the cells were fixed and imaged using confocal fluorescence microscopy.

## Results and discussion

### NTEXPAR probe design

**Fig. 1:**
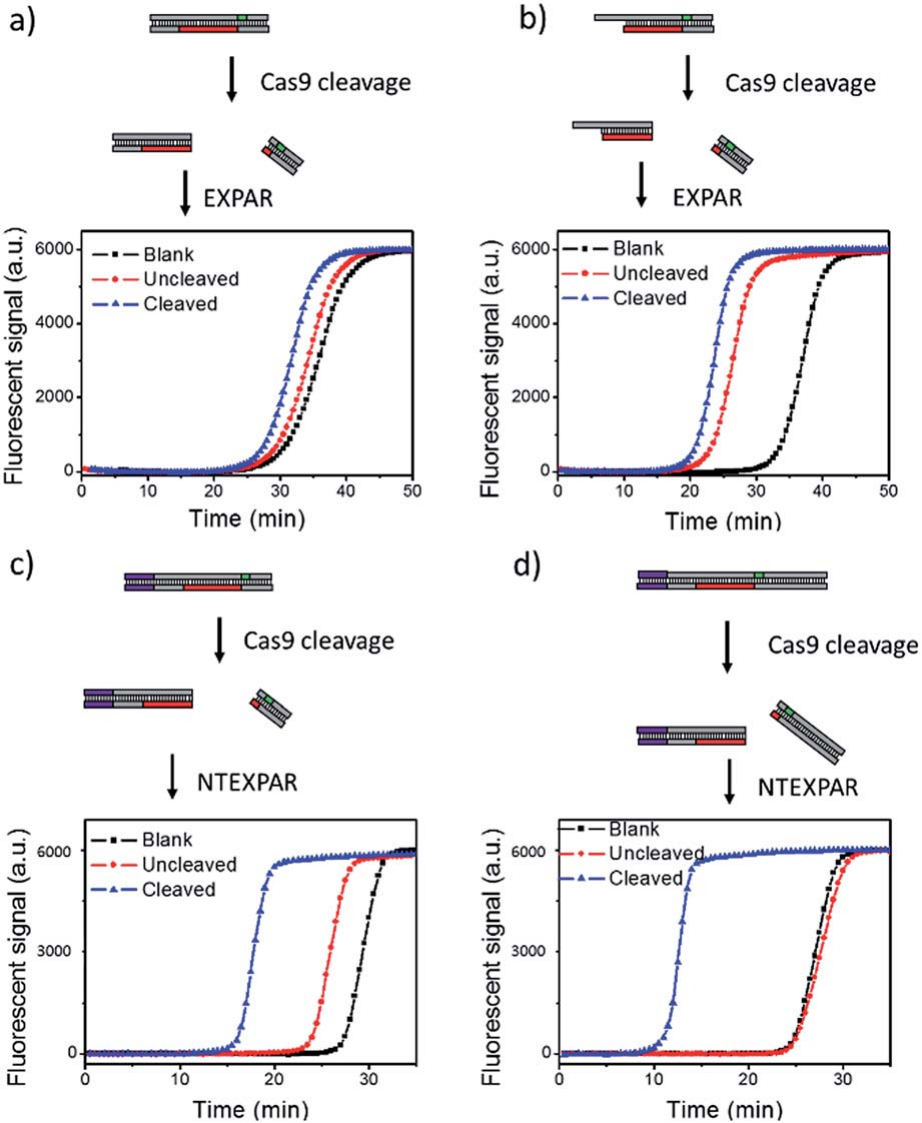
NTEXPAR probe design for Cas9 cleavage assay. (a) EXPAR amplification of 100 pM Cas9 cleaved 37 bp dsDNA substrate (S-substrate and cS-substrate 1). (b) EXPAR amplification of 100 pM Cas9 cleaved 37 bp dsDNA substrate with a truncated complementary sequence (S-substrate and cS-substrate 2). (c) NTEXPAR amplification of 100 pM Cas9 cleaved 60 bp dsDNA substrate (NT-S-substrate and cNT-S-substrate). (d) NTEXPAR amplification of 100 pM Cas9 cleaved 78 bp dsDNA substrate with a nicking site before the target sequence cleaved (NT-l-substrate and cNT-l-substrate). The reaction mechanism of EXPAR and NTEXPAR is illustrated in the ESI (Scheme S1†).

We first tested conventional EXPAR for Cas9 cleaved dsDNA detection (Fig. 1a). Specifically, a 37 base pair (bp) dsDNA with a target sequence and PAM site (S-substrate and cS-substrate 1, see Table S2† for sequence information) was used as the substrate for the cleavage reaction. The cleaved product was then amplified by EXPAR (reaction mechanism illustrated in Scheme S1a†). The EXPAR reaction was monitored by real-time measurement of the fluorescence intensity of the EXPAR products. The uncleaved sample was performed without adding Cas9 nuclease and the blank control was performed without adding dsDNA substrate. When the Cas9 cleaved 37 bp dsDNA was directly used for EXPAR, the real-time fluorescence curve of the cleaved sample showed an insignificant difference to the blank control (Fig. 1a), because of the influence of the complementary sequence. Since the melting temperature (*T*_m_) of the cleaved dsDNA is calculated to be 60.3 °C, the dsDNA hybrid was not able to be unwound to trigger the downstream amplification at the reaction temperature of 55 °C. Therefore, we truncated the complementary sequence to 33-nt (cS-substrate 2) to reduce the calculated *T*_m_ of cleaved dsDNA to 52.5 °C which is below the reaction temperature of 55 °C. After the adjustment, the real-time fluorescence curves of both the uncleaved and the cleaved sample show significant differences from that of the blank control, indicating that the amplification reaction can be triggered by the cleaved dsDNA (Fig. 1b). However, judging from the difference between the uncleaved sample and the blank control, obvious non-specific amplification exists triggered by the uncleaved substrate. It is known that the sensitivity of EXPAR for nucleic acid detection is mainly limited by nonspecific background amplification.^22^ Given these results, EXPAR itself is not suitable for Cas9 cleaved blunt-ended dsDNA detection and a new strategy is needed to dramatically reduce nonspecific amplification.

To solve this issue, we hypothesise that by adding a nicking site before the target sequence, nicking triggered EXPAR (NTEXPAR) could generate single strand DNA triggers for the following amplification reaction and decrease the background signal (the reaction mechanism is illustrated in Scheme S1b†). The major difference between EXPAR and NTEXPAR is that, by adding the nicking site on the target sequence, the Cas9 cleaved dsDNA is able to produce EXPAR triggers which solve the issue of dsDNA detection. To verify this hypothesis, we designed a 60 bp dsDNA substrate with a nicking site (NT-S-substrate and cNT-S-substrate) and used it for Cas9 cleavage analysis. We found that NTEXPAR can efficiently distinguish between uncleaved and cleaved samples (Fig. 1c). However, there was still obvious non-specific amplification and this background signal came from the uncleaved sequence which can accidentally generate the trigger sequence because of its short length. Therefore, we extended the 60 bp sequence to 78 bp and use the longer sequence as the substrate to further suppress the background (Fig. 1d). After extending the dsDNA substrate, the uncleaved and cleaved samples can be obviously distinguished by their real-time fluorescence curves and the uncleaved sample has nearly the same curve as the blank control, which indicates that non-specific amplification is negligible. Therefore, the 78 bp dsDNA was taken as the optimized substrate for the following experiments. Besides the probe optimization, enzyme ratio and concentration were also optimized (Fig. S1 and S2†) and Vent (exo-): 0.05 U μL^–1^, Nt.BstNBI: 0.375 U μL^–1^ were chosen as the optimum conditions for the following experiments.

### NTEXPAR for dsDNA detection

**Fig. 2:**
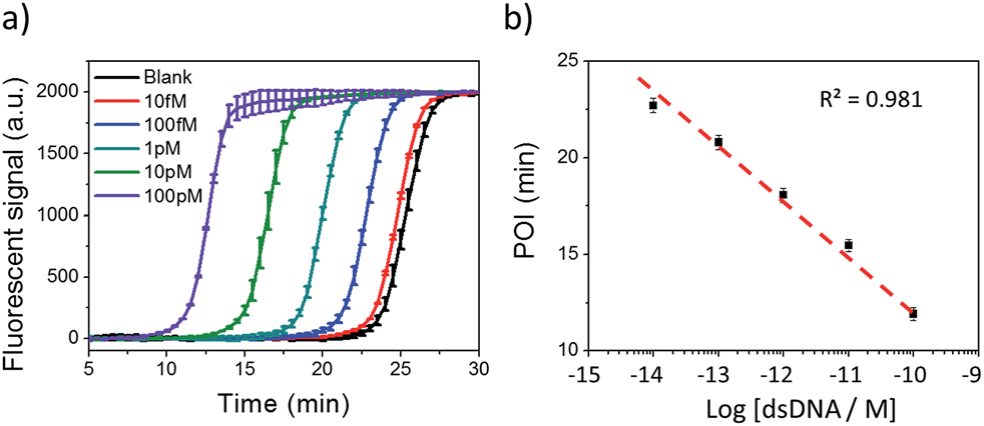
NTEXPAR for Cas9 cleaved dsDNA detection. (a) Real-time fluorescence measurement of NTEXPAR triggered by Cas9 cleaved dsDNA with various concentrations (10 fM to 100 pM). The blank sample contained all the reaction components except for dsDNA. (b) Linear relationship between the point of inflection (POI) values of the corresponding amplification curves and the logarithm of Cas9 cleaved dsDNA concentrations (10 fM to 100 pM). Error bars are based on triplicate experiments.

Under the optimum conditions, Cas9 cleaved dsDNA could be quantitatively detected in a linear range of 10 fM to 100 pM (Fig. 2). The point of inflection (POI), which is defined as the time at which the fluorescence curve rises significantly above the background, was used for quantification.^22^ The POI values linearly correspond to the logarithms of dsDNA concentration with the correlation equation of POI = –14.54 – 2.69 lg[dsDNA] (*R*^2^ = 0.981). To further characterize the NTEXPAR reaction, the amplified products were analyzed by gel electrophoresis (Fig. S3†). The non-specific amplification from the blank control generated products with the same band size of other samples (around 55 bp) which is consistent with the literature^32^ and further proves the reaction mechanism proposed in Scheme 1.

### NTEXPAR for Cas9 cleavage activity analysis

Based on the evidence that NTEXPAR could be used for Cas9 cleaved dsDNA quantification, we next investigated its ability for Cas9 cleavage activity analysis. For this purpose, the dsDNA substrate was cleaved in a 20 μL reaction with 20 nM Cas9 nuclease, 40 nM sgRNA, 1 nM dsDNA substrate and 1 U μL^–1^ Ribolock RNase inhibitor in reaction buffer at 37 °C. A 3.8 kb dsDNA substrate was used here for a head to head comparison between NTEXPAR and gel electrophoresis and we have proved that the amplification performances of NTEXPAR for 78 bp and 3.8 kb dsDNA substrates were not significantly different (Fig. S5†). A time-dependent measurement of Cas9 cleavage was first conducted by withdrawing samples from the reaction at defined time intervals followed by heating at 90 °C to stop the reaction (Fig. S5†). Gel electrophoresis analysis was carried out in parallel with NTEXPAR for comparison. We found that the agarose gel data could not quantify the cleaved dsDNA at different reaction times (Fig. S5a†). On the other hand, the real-time fluorescence curves from NTEXPAR (Fig. S5b†) showed clear differences between reaction times and the percentage of the cleaved dsDNA could be quantified using the standard curve (Fig. 2). By plotting the average percentage of cleaved dsDNA against the reaction time (Fig. S5c†), we found that the Cas9 cleavage reaction was rapid and could be finished within 10 min, which is consistent with the literature.^1^

**Fig. 3:**
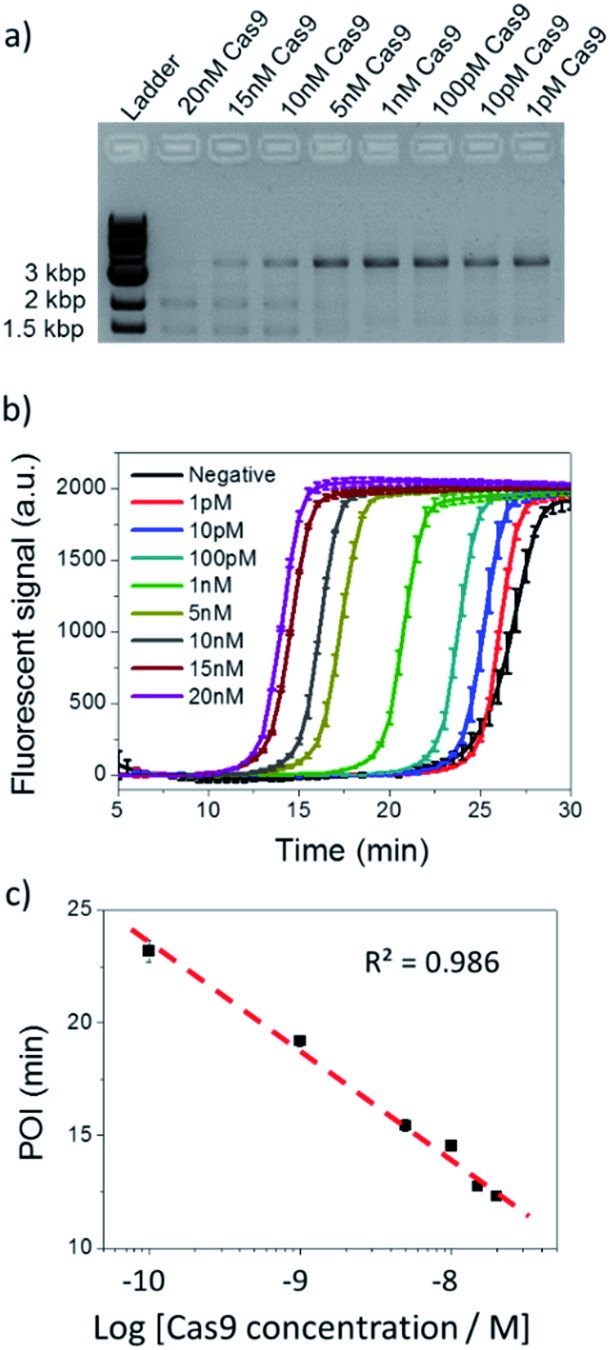
Cas9 cleavage assay with various Cas9 concentrations. (a) Agarose gel electrophoresis analysis of 100 pM dsDNA cleaved by 1 pM to 20 nM Cas9. (b) NTEXPAR analysis of 100 pM dsDNA cleaved by 1 pM to 20 nM Cas9. (c) Linear relationship between the POI values of the corresponding amplification curves and the logarithm of Cas9 concentrations. Error bars are based on triplicate experiments.

Thereafter, the analytical performance of NTEXPAR for Cas9 cleavage analysis was investigated for a series of Cas9 concentrations (Fig. 3). In a typical assay, an *in vitro* Cas9 cleavage reaction was first conducted with a 10 min incubation at 37 °C. Then, 1 μL of reaction mixture was taken out and added to the pre-made NTEXPAR master mix solution which contained all components except the Cas9 cleaved dsDNA. Gel electrophoresis experiments were performed in parallel to compare these two methods (Fig. 3a). As shown in Fig. 3b, the real-time fluorescence signals revealed that, using NTEXPAR, the limit of detection (LOD) of Cas9 was around 10 pM and the detection time could be shortened to ∼40 min (10 min for Cas9 cleavage and 30 min for dsDNA amplification). When the POI values were plotted against the logarithms of Cas9 concentrations (Fig. 3c), the resulting standard curve showed a linear relationship in a range of 100 pM to 20 nM with the correlation equation of POI = –23.95 – 4.74 lg[Cas9 nuclease] (*R*^2^ = 0.986). Compared with NTEXPAR, the agarose gel data could not distinguish the dsDNA cleaved by 1 nM Cas9 nuclease and the enzyme concentration cannot be accurately quantified. In this regard, NTEXPAR could be applied for the sensitive and quantitative analysis of Cas9 cleavage efficiency.

### Pre-screened sgRNA increases Cas9 cleavage specificity

CRISPR/Cas9 activity is modulated by sgRNA sequences and truncated sgRNA has been proven to improve cleavage specificity.^33^ Much research has been conducted to study the effect of sgRNA length. For example, Joung *et al.* proved that the 5′-end nucleotides of sgRNA are not necessary for full sgRNA activity, but normally induce mismatches at other positions.^9^ Other structural and biochemical studies have been conducted to further illustrate the mechanism.^34^–^38^ For instance, using kinetic Monte Carlo (KMC) simulations, Josephs *et al.* showed that Cas9/sgRNA binding specificity is largely determined by the sequence adjacent to PAM. Moreover, Cas9-mediated DNA cleavage specificity is governed by a conformational change of Cas9 nuclease structure that is stabilized by sgRNA interactions at the 14^th^–17^th^ bp region of the protospacer.^39^ Therefore, sgRNA is not necessarily 20-nt. By optimizing sgRNA length, the Cas9 cleavage specificity can be significantly improved.

**Fig. 4:**
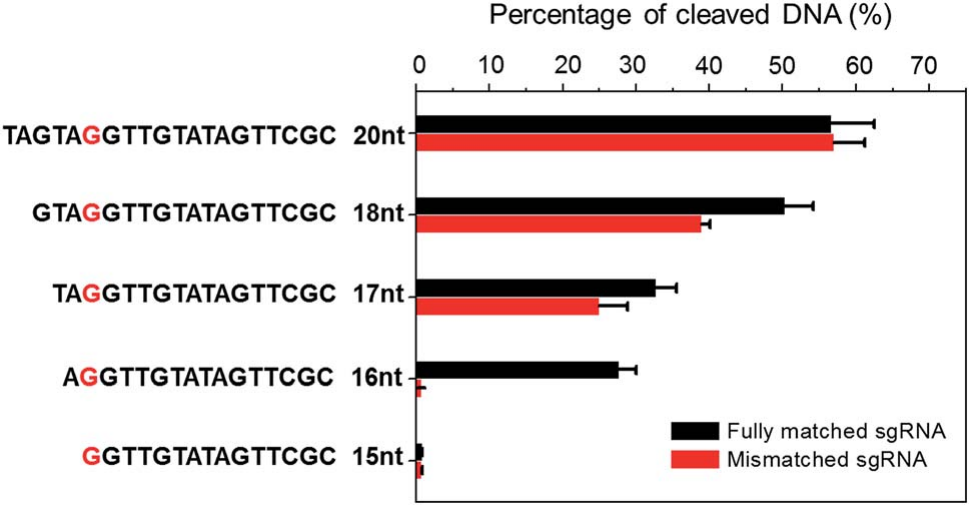
Truncated sgRNA with optimized length enhances Cas9 cleavage specificity. Double stranded DNA substrates were cleaved by Cas9 nuclease with sgRNAs of various lengths (20-nt, 18-nt, 17-nt, 16-nt, and 15-nt). The mismatching site was G/C at the red marked nucleotide. The percentage of Cas9 cleaved dsDNA was measured by NTEXPAR and the concentrations of Cas9 and dsDNA substrate used for all experiments were 2 nM and 100 pM, respectively. Error bars are based on triplicate experiments.

To demonstrate the applicability of NTEXPAR for pre-screening of sgRNA, we performed Cas9 cleavage assays with sgRNAs of 5 different lengths (20-nt, 18-nt, 17-nt, 16-nt, and 15-nt) and each length with a G/C difference at the 15^th^ nucleotide from PAM. The percentage of cleaved dsDNA was measured using NTEXPAR and the quantification data are shown in Fig. 4. When the conventional 20-nt sgRNA was used, there was no obvious cleavage efficiency difference between the fully matched sgRNA and mismatched sgRNA, indicating that the single nucleotide difference at the 15^th^ site could not be distinguished with the classic 20-nt sgRNA. Along with the decrease of sgRNA length, the percentage of cleaved dsDNA also decreased, demonstrating that the cleavage activity was reduced with truncated sgRNA. Importantly, when the sgRNA length was truncated to 16-nt, the mismatched sgRNA suddenly showed nearly no cleavage activity, indicating that the single nucleotide mismatch could be recognized by Cas9 with sgRNA of 16-nt. Given these results, the 16-nt sgRNA could be regarded as the most specific sgRNA for the target sequence.

### Pre-screened sgRNA increase genome editing specificity

**Fig. 5:**
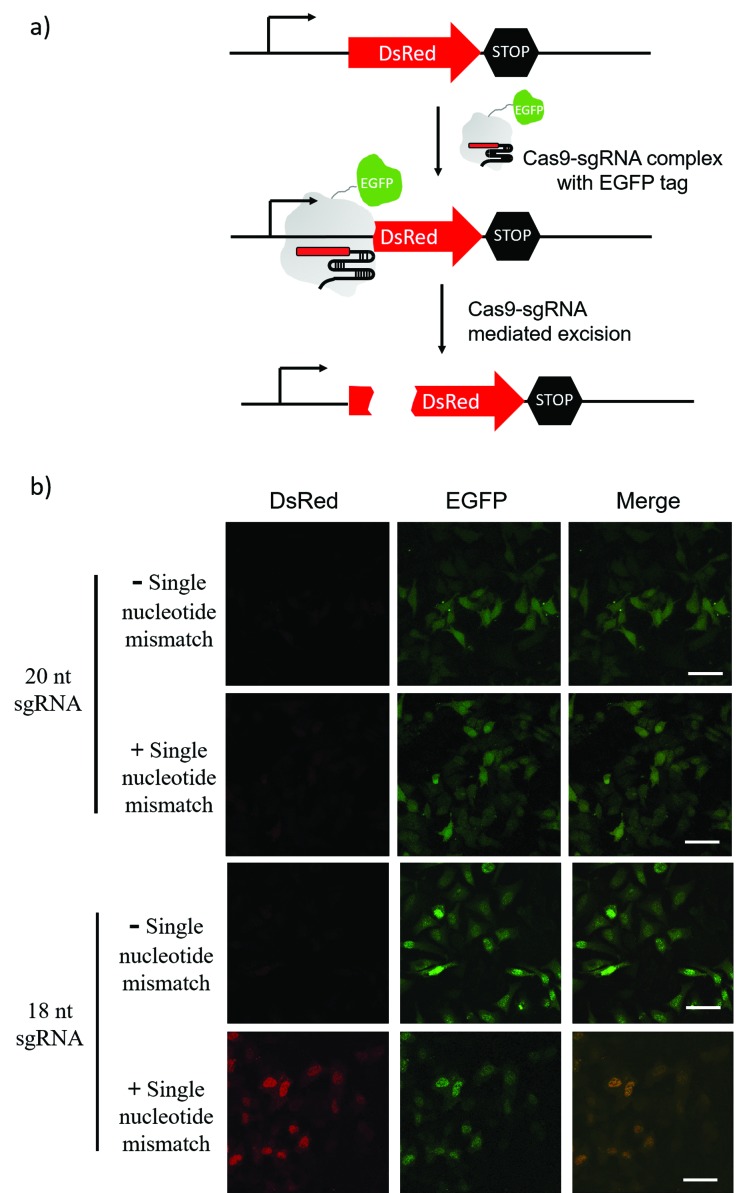
Cas9 mediated DsRed gene silencing using 18-nt sgRNA and 20-nt sgRNA (with or without a single nucleotide mismatch at the 15^th^ nucleotide from PAM). (a) Schematic diagram of Cas9–sgRNA mediated DsRed gene silencing. Cas9 nuclease was labelled with an EGFP tag. In the absence of Cas9, DsRed can be expressed in cells. When the functional Cas9–sgRNA complex was present, the complex mediated excision of the DsRed gene and suppressed DsRed expression. (b) Fluorescence imaging of DsRed-expressing Hela cells transfected with the Cas9/sgRNA expression system and grown for 48 h. Green is EGFP and Red is DsRed. Scale bar is 50 μm.

The sgRNA pre-screening process was then applied to a Cas9 mediated gene silencing experiment to improve the specificity of genome editing. DsRed gene was selected as a fluorescence reporter and the Cas9 nuclease was labelled with an EGFP tag (Fig. 5a). In the absence of Cas9, DsRed could be expressed in cells and gave a red fluorescence signal. When the functional Cas9–sgRNA complexes were present, Cas9 mediated excision of the DsRed gene leaded to suppressed DsRed expression.

Pre-screening of sgRNAs for DsRed gene silencing was first performed with NTEXPAR (Fig. S6†). Based on the quantification results, both 18-nt and 17-nt sgRNAs showed specificity for the G/C mismatch at the 15^th^ nucleotide from PAM, but 18-nt sgRNA presented higher cleavage efficiency. Therefore, the 18-nt sgRNA was chosen as the optimized sgRNA for a cell genome editing application. Cloning 18-nt sgRNAs (with or without a single nucleotide mismatch) into the pSpCas9 (BB) vector for co-expression with Cas9 was performed based on a reported protocol.^40^ DsRed expressing Hela cells were transfected with a Cas9/sgRNA expression system and grown for 48 h. Then, the cells were fixed and imaged using confocal fluorescence microscopy. As shown in Fig. 5b, the conventional 20-nt sgRNA did not have the specificity for the single nucleotide mismatch and suppressed DsRed expression in samples both with or without the single nucleotide mismatch. However, the pre-optimized 18-nt sgRNA was able to distinguish the single nucleotide mismatch and only suppressed DsRed expression in the fully-matched sample. Altogether, these results suggest that pre-screening of sgRNAs could assist in improving the specificity of CRISPR/Cas9 genome editing. Notably, the off-target effects of Cas9 are not only determined by the cleavage specificity, the competition of similar DNA sequences in a genome context also induces unwanted off-target effects which cannot be reduced by sgRNA optimization. To address this issue, a computer assisted design is needed to first choose candidate sequences that have minimal similar sequences in genome.

## Conclusions

In summary, we have developed a novel Cas9 cleavage assay using a nicking triggered exponential amplification reaction (NTEXPAR) for quantitative evaluation of Cas9 cleavage efficiency and pre-screening of sgRNA to achieve highly specific and highly efficient CRISPR/Cas9 genome editing. By real-time measurement of fluorescence intensity, a dsDNA substrate cleaved by down to 10 pM Cas9 can be accurately detected within 40 min. We have applied the pre-screened sgRNA in a gene silencing experiment and find that it can significantly improve genome editing specificity to a single nucleotide mismatch level. Compared with gel electrophoresis analysis, this fast and cost-effective sgRNA pre-screening approach has the potential to assist in the design of genome editing strategies.

## Supplementary Material

Supplementary information
