# Supplementary material for: Cas9 cleavage assay for pre-screening of sgRNAs using nicking triggered isothermal amplification
Source: Chem Sci. 2016 Apr 29;7(8):4951–7. doi: 10.1039/c6sc01355d (PMC6018437; doi:10.1039/c6sc01355d)
Supplement: Supplementary file 1 [file SC-007-C6SC01355D-s001.pdf]

Electronic Supplementary Material (ESI) for Chemical Science.
This journal is © The Royal Society of Chemistry 2016

Supporting Information

Cas9 Cleavage Assay for Pre-screening of sgRNAs using Nicking Triggered Isothermal Amplification

*Kaixiang Zhang, Ruijie Deng, Yue Li, Ling Zhang, Jinghong Li**

Department of Chemistry, Analysis Center, Tsinghua University, Beijing 100084, China
E-mail: jhli@mail.tsinghua.edu.cn

1. Supplementary Tables

Table S1. Primers used for sgRNA template generation (for *in vitro* transcription).

Name	Sequence
R-Primer	5'-AGCACCGACTCGGTGCCACTT-3'
F-primer 20nt	5'- TAA TAC GAC TCA CTA TAG TAG TAG GTT GTA TAG TTC GC G TTT TAG AGC TAG AAA TAG C -3'
F-primer 18nt	5'- TAA TAC GAC TCA CTA TAG G TAG GTT GTA TAG TTC GC G TTT TAG AGC TAG AAA TAG C -3'
F-primer 17nt	5'- TAA TAC GAC TCA CTA TAG TAG GTT GTA TAG TTC GC G TTT TAG AGC TAG AAA TAG C -3'
F-primer 16nt	5'- TAA TAC GAC TCA CTA TAG AG GTT GTA TAG TTC GC G TTT TAG AGC TAG AAA TAG C -3'
F-primer 15nt	5'- TAA TAC GAC TCA CTA TAG G GTT GTA TAG TTC GC G TTT TAG AGC TAG AAA TAG C -3'
F-primer 14nt	5'- TAA TAC GAC TCA CTA TAG GTT GTA TAG TTC GC G TTT TAG AGC TAG AAA TAG C -3'
F-primer M-20nt	5'- TAA TAC GAC TCA CTA TAG TAG TAC GTT GTA TAG TTC GC G TTT TAG AGC TAG AAA TAG C -3'
F-primer M-18nt	5'- TAA TAC GAC TCA CTA TAG G TAC GTT GTA TAG TTC GC G TTT TAG AGC TAG AAA TAG C -3'
F-primer M-17nt	5'- TAA TAC GAC TCA CTA TAG TAC GTT GTA TAG TTC GC G TTT TAG AGC TAG AAA TAG C -3'
F-primer M-16nt	5'- TAA TAC GAC TCA CTA TAG AC GTT GTA TAG TTC GC G TTT TAG AGC TAG AAA TAG C -3'
F-primer M-15nt	5'- TAA TAC GAC TCA CTA TAG C GTT GTA TAG TTC GC G TTT TAG AGC TAG AAA TAG C -3'

Table S2. Oligonucleotides used for DNA substrate optimization.

Name	Sequence
S-substrate	5'- TGA GGT AGT AGG TTG TAT AGT T CGC TGG AGT ACA AAC -3'
cS-substrate 1	5'- GTT TGT ACT CCA GCG AAC TAT ACA ACC TAC TA C CTC A -3'
cS-substrate 2	5'- GTT TGT ACT CCA GCG AAC TAT ACA ACC TAC TAC-3'
NT-S-substrate	5'-CAG CTA GAGTC G AAG TAG TGA TT T GAG GTA GTA GGT TGT ATA GTT CGC TGG AGT ACA AAC -3'
cNT-S-substrate	5'- GTT TGT ACT CCA GCG AAC TAT ACA ACC TAC TAC CTC AAA TCA CTA CTT C GACTC TAG CTG -3'
NT-L-substrate	5'-CAG CTA GAGTC G AAG TAG TGA TT T GAG GTA GTA GGT TGT ATA GTT CGC TGG AGT ACA AAC GTC AGC TGT TTT AGA GCT -3'
cNT-L-substrate	5'- AGC TCT AAA ACA GCT GAC GTT TGT ACT CCA GCG AAC TAT ACA ACC TAC TAC CTC AAA TCA CTA CTT C GACTC TAG CTG -3'
EXPAR template	5'-A ACT ATA CAA CCT ACT ACC TCA AACA GACTC A AA CTA TAC AAC CTA CTA CCT CAA-3'

* Target sequence in red, protospacer-adjacent motif (PAM) in green, nicking site in purple

and EXPAR template recognition sequence in blue.

Table S3. Oligonucleotides used for sgRNA pre-screening for DsRed gene silencing.

Name	Sequence
DsRed substrate	5'-CAG CTA GAGTC G AAG TAG TGA TT ACG GTG TTG TGG CCC TC GTA GGG AGT ACA AAC GTC AGC TGT TTT AGA GCT -3'
cDsRed substrate	5'- AGC TCT AAA ACA GCT GAC GTT TGT ACT CCC TAC GAG GGC CAC AAC ACC GT A ATC ACT ACT TC GACTC TA GCT G -3'
Cleaved DsRed	5'-CAG CTA GAGTC G AAG TAG TGA TT ACG GTG TTG TGG CCC TC-3'
cCleaved DsRed	5'- GAG GGC CAC AAC ACC GTA ATC ACT ACT TC GACTC TA GCT G -3'
DsRed template	5'-GAG GGC CAC AAC ACC AACA GACTC A GAG GGC CAC AAC ACC-3'

*Target sequence in red, protospacer-adjacent motif (PAM) in green, nicking site in purple and EXPAR template recognition sequence in blue.

Table S4. Primers used for sgRNA template generation (for *in vitro* transcription).

Name	Sequence
DsRed R-Primer	5'-AGCACCGACTCGGTGCCACTT-3'
DsRed F-primer 20nt	5'- TAA TAC GAC TCA CTA TAG <u>ACG GTG TTG TGG CCC TC GTA</u> G TTT TAG AGC TAG AAA TAG C -3'
DsRed F-primer 18nt	5'- TAA TAC GAC TCA CTA TAG <u>G GTG TTG TGG CCC TC GTA</u> G TTT TAG AGC TAG AAA TAG C -3'
DsRed F-primer 17nt	5'- TAA TAC GAC TCA CTA TAG <u>GTG TTG TGG CCC TC GTA</u> G TTT TAG AGC TAG AAA TAG C -3'
DsRed F-primer 16nt	5'- TAA TAC GAC TCA CTA TAG <u>TG TTG TGG CCC TC GTA</u> G TTT TAG AGC TAG AAA TAG C -3'
M-DsRed F-primer 20nt	5'- TAA TAC GAC TCA CTA TAG <u>ACG GTC TTG TGG CCC TC GTA</u> G TTT TAG AGC TAG AAA TAG C -3'
M-DsRed F-primer 18nt	5'- TAA TAC GAC TCA CTA TAG <u>G GTC TTG TGG CCC TC GTA</u> G TTT TAG AGC TAG AAA TAG C -3'
M-DsRed F-primer 17nt	5'- TAA TAC GAC TCA CTA TAG <u>GTC TTG TGG CCC TC GTA</u> G TTT TAG AGC TAG AAA TAG C -3'
M-DsRed F-primer 16nt	5'- TAA TAC GAC TCA CTA TAG <u>TC TTG TGG CCC TC GTA</u> G TTT TAG AGC TAG AAA TAG C -3'

Table S5. Sequences used for sgRNA insertion

Name	Sequence
DsRed 20nt top	5'-CACC G ACG GTG TTG TGG CCC TCG TA-3'
DsRed 20nt bottom	5'-AAAC TA CGA GGG CCA CAA CAC CGT C-3'
M-DsRed 20nt top	5'-CACC G ACG GTC TTG TGG CCC TCG TA-3'
M-DsRed 20nt bottom	5'-AAAC TA CGA GGG CCA CAA GAC CGT C-3'
DsRed 18nt top	5'-CACC G G GTG TTG TGG CCC TCG TA-3'
DsRed 18nt bottom	5'-AAAC TA CGA GGG CCA CAA CAC C C-3'
M-DsRed 18nt top	5'-CACC G G GTC TTG TGG CCC TCG TA-3'
M-DsRed 18nt bottom	5'-AAAC TA CGA GGG CCA CAA GAC C C-3'

2. Schematic diagram of EXPAR and NTEXPAR

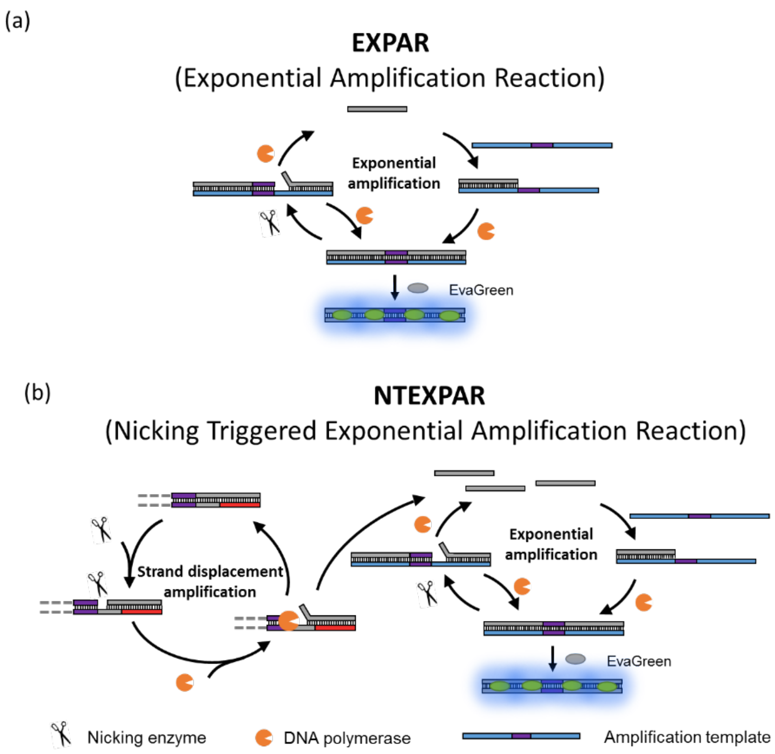

Scheme S1. Mechanism of EXPAR (a) and NTEXPAR (b). The major difference is that NTEXPAR can be used for double strand DNA detection (dsDNA). By adding the nicking site on the target sequence, the Cas9 cleaved dsDNA can be used for triggering downstream isothermal amplification.

3. Enzyme ratio and concentration optimization

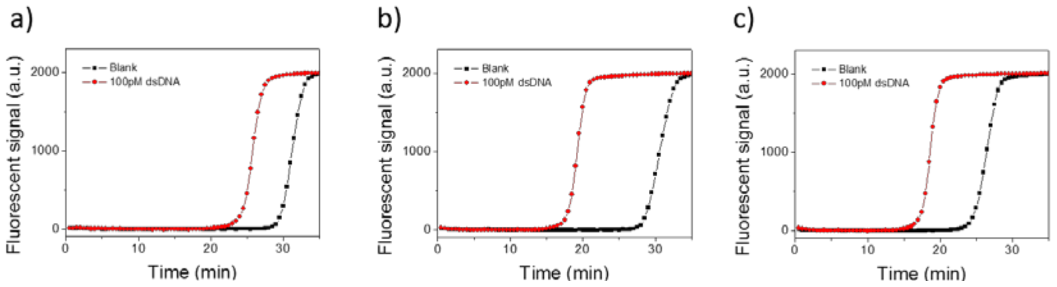

Figure S1. The influence of enzyme ratio on NTEXPAR. The real-time fluorescence curves were produced by 100 pM Cas9 cleaved dsDNA. a) Vent (exo$^-$): 0.033 U/μL, Nt. BstNBI: 0.165 U/μL, ΔPOI=4.42. b) Vent (exo$^-$): 0.033 U/μL, Nt. BstNBI: 0.248 U/μL, ΔPOI=10.94. c) Vent (exo$^-$): 0.033 U/μL, Nt. BstNBI: 0.333 U/μL, ΔPOI=6.60.

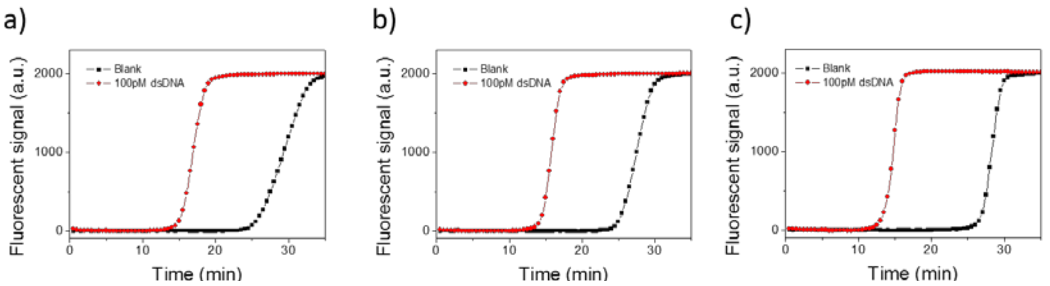

Figure S2. The influence of enzyme concentration on NTEXPAR. The real-time fluorescence curves were produced by 100 pM Cas9 cleaved dsDNA. a) Vent (exo$^-$): 0.017 U/μL, Nt. BstNBI: 0.124 U/μL, ΔPOI=10.32. b) Vent (exo$^-$): 0.033 U/μL, Nt. BstNBI: 0.248 U/μL, ΔPOI=10.82. c) Vent (exo$^-$): 0.05 U/μL, Nt. BstNBI: 0.375 U/μL, ΔPOI=12.53.

4. Gel analysis of NTEXPAR product

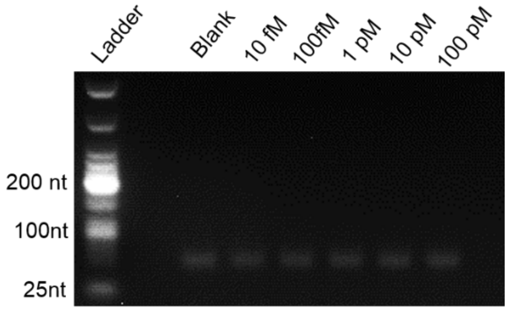

Figure S3. Agarose gel electrophoresis analysis of NTEXPAR product amplified from different dsDNA concentration after 1 h reaction. Blank sample contained all reaction components except the dsDNA target.

5. NTEXPAR analysis of short and long dsDNA

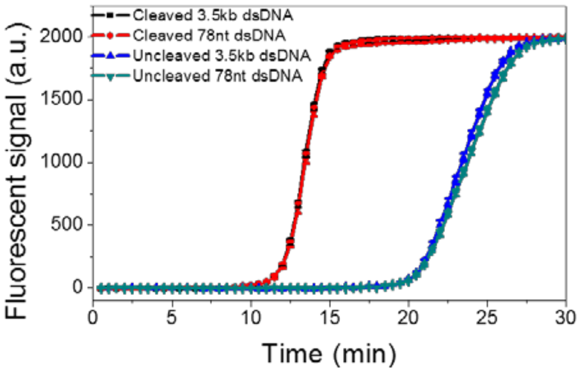

Figure S4. NTEXPAR analysis of Cas9 cleaved and uncleaved short (78-nt) and long (3.5 kb) dsDNA. Error bar is based on triplicate experiments.

6. Cas9 cleavage analysis in a time-dependent manner

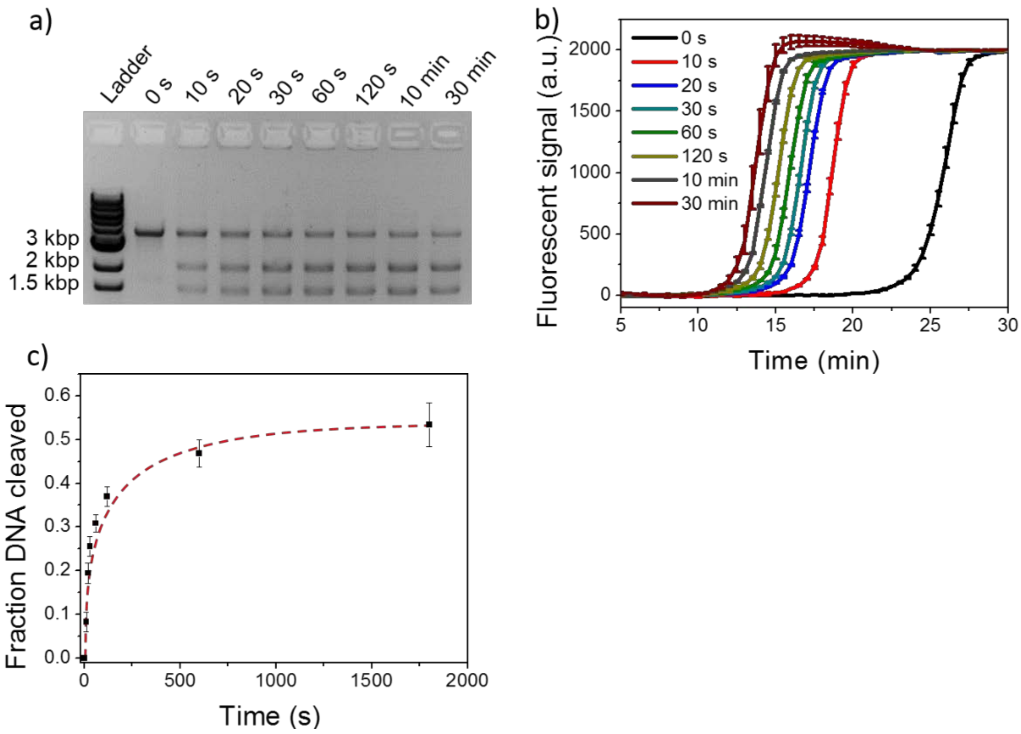

Figure S5. Cas9 cleavage analysis in a time-dependent manner. a) Agarose gel electrophoresis analysis of 100 pM Cas9 cleaved dsDNA at different reaction time. b) NTEXPAR analysis of 100 pM Cas9 cleaved dsDNA at different reaction time. Error bars are based on triplicate experiments. c) The average percentage of Cas9 cleaved dsDNA (calculated by NTEXPAR) is plotted vs. the reaction time.

7. Per-screening of sgRNA for DsRed gene silencing

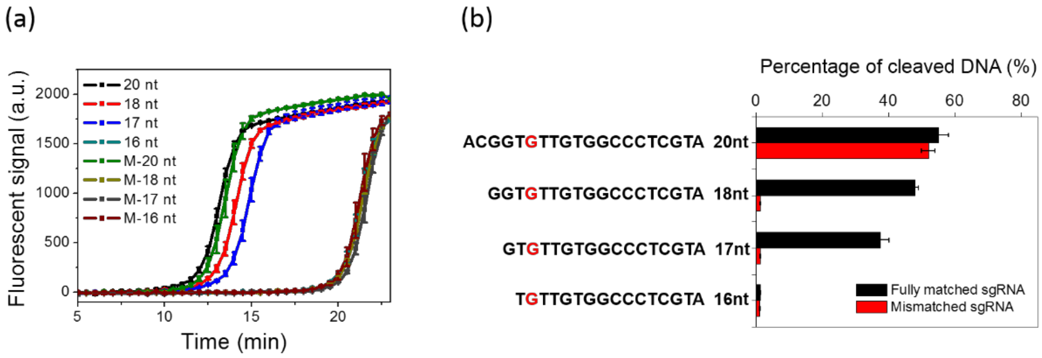

Figure S6. Per-screening of sgRNA for DsRed gene silencing. Single guide RNAs of various lengths (20-nt to 16-nt) and mismatching site (marked in red) were used for screening. a) Real-time fluorescent signal of NTEXPAR for sgRNA pre-screening. b) The percentage of cleaved DNA calculated by NTEXPAR. Both 18-nt and 17-nt sgRNA showed specificity for dsDNA substrate cleavage. Error bars are based on triplicate experiments.
